# Efficacy and acceptability of selective serotonin reuptake inhibitors for the treatment of depression in Parkinson's disease: a systematic review and meta-analysis of randomized controlled trials

**DOI:** 10.1186/1471-2377-10-49

**Published:** 2010-06-21

**Authors:** Petros Skapinakis, Eleni Bakola, Georgia Salanti, Glyn Lewis, Athanasios P Kyritsis, Venetsanos Mavreas

**Affiliations:** 1 Department of Psychiatry, University of Ioannina School of Medicine, Ioannina 45110, Greece; 2 Academic Unit of Psychiatry, University of Bristol, Cotham House, Cotham Hill, Bristol BS66JL, UK; 3 Department of Hygiene and Epidemiology, University of Ioannina School of Medicine, Ioannina 45110, Greece; 4 Department of Neurology, University of Ioannina School of Medicine, Ioannina 45110, Greece

## Abstract

**Background:**

Selective serotonin reuptake inhibitors (SSRIs) are the most commonly prescribed antidepressants for the treatment of depression in patients with Parkinson's Disease (PD) but data on their efficacy are controversial.

**Methods:**

We conducted a systematic review and meta-analysis of randomized controlled trials to investigate the efficacy and acceptability of SSRIs in the treatment of depression in PD.

**Results:**

Ten studies were included. In the comparison between SSRIs and Placebo (n = 6 studies), the combined risk ratio (random effects) was 1.08 (95% confidence interval: 0.77 - 1.55, p = 0.67). In the comparison between SSRIs and Tricyclic Antidepressants (TCAs) (n = 3 studies) the combined risk ratio was 0.75 (0.39 - 1.42, p = 0.37). An acceptability analysis showed that SSRIs were generally well tolerated.

**Conclusions:**

These results suggest that there is insufficient evidence to reject the null hypothesis of no differences in efficacy between SSRIs and placebo in the treatment of depression in PD. Due to the limited number of studies and the small sample sizes a type II error (false negative) cannot be excluded. The comparison between SSRIs and TCAs is based on only three studies and further trials with more pragmatic design are needed.

## Background

Major depressive disorder is common among patients with Parkinson's disease (PD). A recent systematic review reported that the prevalence of depression may range from 8% in community-based patients to more than 20% in outpatient or inpatient settings, while depressive symptoms are even more common [1]. The impact of depression in the quality of life of patients with PD has been recently recognized even in community-based patients and is independent of disease severity and other clinical or demographic variables [2,3]. Depression is also associated with increased mortality in PD patients [4] and is the most important risk factor for suicide especially after neurosurgical treatment of PD [5]. Thus, recognizing and treating depression in the context of PD is important to reduce disability and improve prognosis.

Treatment of depression with antidepressant drugs is well established. In the last 20 years use of antidepressant has risen mainly due to the introduction of the selective serotonin reuptake inhibitors [SSRIs). These drugs are now the most commonly prescribed antidepressants in patients with depression in general [6]. Regarding depression in the context of PD, a recent survey in the U.S. showed that 63% of the prescriptions for depression in PD were for SSRIs and only 7.5% for tricyclic antidepressants (TCAs) [7]. The preference of SSRIs over the older TCAs is supposedly based on their similar efficacy but better tolerability, especially when compared with tertiary amines, such as amitriptyline or imipramine [8].

Treatment of depression in the context of PD (and other medical illnesses) poses, however, particular problems: a) most antidepressant trials exclude patients with comorbid medical illnesses and therefore their results cannot be generalized to these patients, b) diagnosis and assessment of severity of depression in patients with PD may be more difficult because of overlapping symptoms and the use of depression rating scales that were not specifically designed to assess depression in this context [9], c) trials that specifically aim to investigate the efficacy of antidepressants in depression comorbid with a medical illness are usually carried out by independent researchers and often are small and based on single centres.

Given these difficulties it is important to systematically review all available evidence regarding the efficacy of SSRIs in depression in the context of PD and if possible to carry out a quantitative synthesis. Previous meta-analyses of the efficacy of antidepressants in the context of PD did not specifically focus on SSRIs [10-12]. Moreover, even the most recent meta-analysis [13] only included two SSRI trials with the latest being published in 2003 [14]. This review concluded that SSRIs were associated with a negligible effect size of 0.05 compared to placebo [95% confidence interval: -0.64, 0.75) but this result was only based on 32 randomized patients and the analysis was clearly underpowered for such a comparison [13]. Since then, several new trials have been published comparing SSRIs with placebo or other comparator interventions and a new meta-analysis is justified given the small sample size of most trials.

The aim of this paper was therefore to systematically review all randomized controlled trials that studied the efficacy of SSRIs in treating depression in the context of PD. Our primary aim was to compare the antidepressant response in SSRIs versus placebo by carrying out a meta-analysis of all randomized trials. A secondary aim was to compare SSRIs versus TCAs if the number of trials identified would allow such a comparison. We finally aimed to examine the safety and tolerability of the use of SSRIs in this patient group.

## Methods

### Search strategy

We searched PubMed for English and non-English medical literature published from 1966 to December 2008. We supplemented this source by also searching EMBASE (1980 - 2008), the Cochrane Controlled Trials Register (2008, issue 4) and the PsiTri database http://psitri.stakes.fi/. We also searched for trials in progress or completed in http://www.clinicalTrials.gov. We manually checked the reference lists of prior reviews, systematic reviews and trials.

We used the following search string (string 1) in PubMed: (serotonin uptake inhibitors OR SSRI* OR citalopram OR escitalopram OR paroxetine OR fluvoxamine OR fluoxetine OR sertraline OR clomipramine OR venlafaxine OR duloxetine) AND (parkinson* OR parkinsoni*). Because the use of the MeSH terms does not return records that have been supplied by the publishers or are in the process of indexing, we also used the following sensitive string (string 2) to identify new studies not yet officially indexed: (serotonin inhibit* OR SSRI* OR citalopram OR escitalopram OR paroxetine OR fluvoxamine OR fluoxetine OR sertraline OR clomipramine OR venlafaxine OR duloxetine) AND (parkinson* OR parkinsoni*) AND (publisher [sb] OR (in process [sb])).

Additional strategy for identifying trials included searching the reference list of the retrieved studies.

### Inclusion and exclusion criteria

Studies included in our systematic review were required to meet all the following criteria:

• Study design: randomised controlled trial.

• Participants: Study participants were required to have a clinical diagnosis of idiopathic Parkinson's disease and also a clinical diagnosis of depression (as defined by the authors of the trials). Both gender and all ages are included.

• Pharmacological intervention: Included studies were required to have at least one arm in which an SSRI was given as the main treatment. Acceptable comparator groups included placebo or other antidepressant treatment such as other antidepressant medications or other biological or psychological treatments (e.g. electroconvulsive therapy, repetitive transcranial magnetic stimulation (rTMS) or cognitive behavioural therapy).

• Outcome measurement: assessment of the change in the score of the depression rating scale used in each study and/or assessment of the response to the treatment as defined in each study.

We excluded studies from our review if they met the following criterion:

• Depression was not assessed with a validated instrument (e.g. Hamilton Depression Rating Scale, Beck Depression Inventory e.t.c.).

In addition to these criteria, in our quantitative synthesis (meta-analysis) we also excluded studies that met the following criteria:

• The study did not report a binary outcome (response vs non-response) or such an outcome was not possible to be extracted from the paper or by directly contacting the authors of the study.

• The comparator was not placebo or a drug officially licensed for the treatment of depression.

### Data extraction, outcome measurement and assessment of methodological quality

Data extracted included information on:

a) the authors, the country, the publication year;

b) patients (age, sex, depression diagnosis, PD diagnosis, duration and stage of PD);

c) methods (study design, depression scale used, definition of treatment response, duration of study);

d) interventions (type and dose of SSRI used, type and/or dose of the control intervention);

e) outcomes and results (number of patients entering and completing the study, number and reasons for dropouts and withdrawals, number of patients responding in active and control arms).

Our primary outcome measure was the number of patients in each treatment group who responded to treatment. Response was defined as the proportion of patients who had a reduction of at least 50% from the baseline score on the Hamilton Depression Rating Scale (HDRS) or the Montgomery-Åsberg Depression Rating Scale (MADRS) or who scored much or very much improved in the Clinical Global Impression Scale (CGI). When a trial had reported results from several scales, we used the HDRS as the first choice, followed by MADRS and CGI. We used the dichotomous response as our primary outcome and not reduction in the severity of symptoms measured as a continuous outcome, because we think that results are more readily interpretable from a clinical perspective. Although, the focus of this review was the efficacy of SSRIs, we also measured the total number of dropouts in each arm to assess the acceptability of these drugs in Parkinson's disease patients with depression.

Data extraction was performed independently by two of the authors (PS, EB) and checked by another (VM). In case of disagreement two senior authors (PS, VM) reviewed the studies and reached a consensus.

To assess the methodological quality of included trials we used the criteria for quality assessment recommended by the Cochrane Collaboration Handbook [15] which are mainly focused on descriptions of sequence generation, allocation concealment, blinding, completeness of outcome data, selective outcome reporting and other potential sources of bias.

### Statistical Analysis

Data from the data sheets were entered into Review Manager version 4.2 [16] by two investigators using the duplicate data entry facility of the software. Number of respondents in each study were recorded according to the intention to treat principle and this was based on the total number of patients randomized to each treatment. For the quantitative synthesis and the generation of forest plots we also used "Comprehensive meta-analysis" version 2.0 http://www.meta-analysis.com. Using the latter we calculated risk ratios for antidepressant response (ratios of the number of patients who responded divided by the number of patients initially randomized to the respective group) and their 95% confidence intervals. Risk ratios greater than 1 indicate a better response for the SSRI group. To investigate the degree of between-trial heterogeneity we used the Q and I-squared statistics [17]. In the presence of significant heterogeneity the potential sources were investigated (type of antidepressant, patient characteristics, study quality). As we anticipated that most of the included studies would have small sample sizes we did not base our choice of modelling upon the heterogeneity statistics. Instead, the random effects model was a priori selected to combine risk ratios as this method is more robust and has better external generalisability. Our primary research interest was to compare the efficacy of SSRIs as a class versus placebo. Our secondary aim was to compare the efficacy of SSRIs versus TCAs if the number of trials and sample size would allow such a comparison. Side effects are presented in a descriptive way but we additionally extracted the total number of those who dropped out of the study for any reason (including those who left due to side effects) from each study and we present this analysis in the text. We used the total number of dropouts and not the number of dropouts due to side effects because of the small sample size.

## Results

### Search strategy results

**Figure 1:**
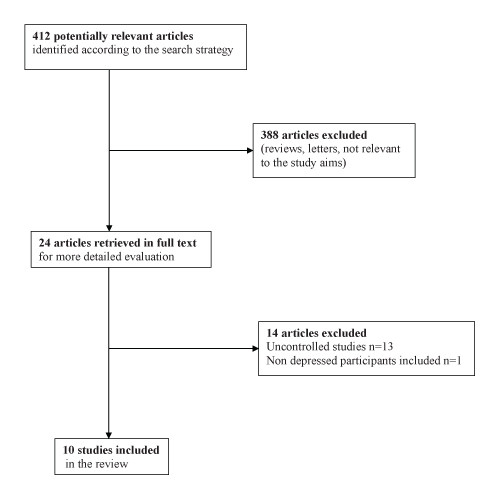
**Flow diagram of the Study**.

The results of our search strategy are presented in Figure 1. A total of 412 potentially relevant articles were identified according to our search strategy and other sources (reference list of previous systematic reviews or retrieved papers). We excluded 388 papers because they were reviews, letters or irrelevant to the study aims. We retrieved the full text of 24 articles for a more detailed evaluation and 14 of them were excluded: 13 studies were uncontrolled; one study - [18] - randomized PD patients to citalopram or placebo only if they were non-depressed while the depressed participants did not have a control group (see Additional file 1 Table s1 for a list of studies excluded from the review). Finally 10 studies were included in the review.

### Description of studies

The characteristics of the ten trials are summarized in table 1. These trials included three sertraline studies (10 to 12 week duration) [14,19,20], two citalopram studies (4 to 6 week duration) [21,22], three fluoxetine studies (8 weeks to 3 months duration) [23-25], one paroxetine study (8 weeks duration) [26] and one fluvoxamine study (16 week duration) [27].

**Table 1:** Characteristics of the randomized controlled trials included in the systematic review and meta-analysis

Study/Country	SSRI/dosage	Comparator	N (% male)	Mean age (age range or SD)	Duration of study	Mean duration of PD (SD)	Hoehn and Yahr stage mean (SD)	Depression Scale	Treatment responders (response rate)	Dropouts
Devos et al. (2008)/France [22]	citalopram (20 mg/day)	1. placebo 2. desipramine (75 mg/day)	48 (NA)	61.8 (56-68)	4 weeks	8 years	NA	MADRS	citalopram: 8/15 (53%) placebo: 4/16 (25%) desipramine: 11/17 (65%)	3
Wermuth et al. (1998)/Denmark [21]	citalopram (10-20 mg/day)	placebo	37 (43%)	64 (44-79)	6 weeks (acute phase)	NA	I-III (range)	HDRS	citalopram: 2/18 (11%) placebo: 3/19 (16%)	7
Antonini et al. (2006)/Italy [19]	sertraline (50 mg/day)	amitriptyline (25 mg/day)	31 (45%)	S: 71.8 (6.5) ^a ^C: 68.5 (6.6) ^a^	12 weeks	S: 7.5 years (3.4) ^a ^C: 7.3 years (4.5) ^a^	S: 2.0 ( 0.7) ^a ^C: 2.4 (0.6) ^a^	HDRS	sertraline: 10/16 (63%) amitriptyline: 8/15 (53%)	8
Barone et al. (2006)/Italy [20]	sertraline (mean dose: 48.1 ± 5.9 mg/day)	pramipexole (mean dose: 3.24 ± 1.3 mg/day)	67 (52%)	S: 68.1 (6.5) C: 64.8 (8.3)	12 weeks	NA	S: 2.5 (median) C: 2 (median)	HDRS	sertraline: 16/34 (47%) pramipexole: 23/33 (70%)	8
Leentjens et al. (2003)/The Netherlands [14]	sertraline (25-100 mg/day)	placebo	12 (67%)	67 (7.8)	10 weeks	NA	I-IV (range)	MADRS	sertraline: 3/6 (50%) placebo: 4/6 (67%)	0
Fregni et al. (2004)/USA [25]	fluoxetine (20 mg/day)	rTMS (15 Hz)	43 (62%) ^a^	S: 66.0 (8.5) ^a ^C: 65.3 (7.8) ^a^	8 weeks	NA	S: 2.1 (1.2) ^a ^C: 2.1 (1.2) ^a^	HDRS	fluoxetine: 9/21 (43%) rTMS: 9/22 (41%)	1
Serrano-Duenas (2002)/Ecuador [23]	fluoxetine (mean dose: 27.3 mg/day)	amitriptyline (mean dose: 35.2 mg/day)	77 (56%)	68.2 (4.5)	12 months	6.9 years (0.8)	II	HDRS	NA	19
Avila et al. (2003)/Spain [24]	fluoxetine (mean dose: 25 mg/day)	nefazodone (mean dose: 200 mg/day)	16 (44%)	70.4 (59-78)	12 weeks	5 years	S: 2.6 (0.8) C: 2.3 (0.5)	BDI	NA	3
Menza et al. (2008)/USA [26]	paroxetine CR (mean dose: 28.4 mg/day)	1. placebo 2. nortriptyline (mean dose: 48.5 mg/day)	52 (52%)	62.2 (8.7)	8 weeks	6.6 years	2.2	HDRS	paroxetine: 2/18 (11%) placebo: 4/17 (24%) nortriptyline 9/17 (53%)	18
Rabey et al. (1996)/Israel [27]	fluvoxamine (mean dose: 78 mg/day)	amitriptyline (mean dose: 69 mg/day)	47 (NA)	75 (NA)	16 weeks	7 years	NA	HDRS	fluvoxamine: 12/20 (60%) amitriptyline: 15/27 (56%)	20

PD: Parkinson's disease; MADRS: Montgomery-Asberg Depression Rating Scale; HDRS: Hamilton Depression Rating Scale; BDI: Beck Depression Inventory S: SSRI- group; C: comparator-group; rTMS: repetitive transcranial magnetic stimulation; SD: standard deviation; NA: not available

^a ^Demented patients are excluded; ^b ^available data only for complete

The mean age of the participants was over 65 years for the majority of the studies. In most studies participants were diagnosed with idiopathic Parkinson's disease according to the United Kingdom's Parkinson's Disease Society Brain Bank (UK-PDS-BB). In most studies depression was diagnosed according to Diagnostic and Statistical Manual of Mental Disorders 3^rd ^revised or 4^th ^edition (DSM-III-R or DSM-IV) criteria [28]. Five studies included patients with major depression only [14,19-22], one study enrolled patients with major or minor depression [25], two studies included patients with major depression or dysthymic disorder [24,26] and two studies included patients with a depression diagnosis without specifying further [23,27].

In studies that used a dichotomous outcome (response) this was defined as an at least 50% reduction from the baseline score in the depression scale used. Most studies used the Hamilton Depression Rating Scale (HDRS) to assess the severity of depression and treatment response [19-21,23,25-27]. Mean baseline scores of the HDRS were between 19-21 for most of the studies, corresponding to moderate depression. One study [25] had baseline scores of more than 25, indicating severe depression and one study had unusually high scores >40 [23]. Montgomery-Åsberg Depression Rating Scale (MADRS) was used in two studies [14,22], with mean baseline scores of 19-20 in one study [14] and a median of >25 in the second study [22]. Other assessment tools for depression included Beck Depression Inventory (BDI) [24,25], Melancholia Scale (MES) [21] and Zung-Self Rating Depression Scale (SDS) [20]. Clinical Global Impression Scale (CGI) was additionally used in three studies [21,24,26]. For the neurological evaluation of the participants the studies used Hoehn and Yahr scale [14,19-21,23-26,29] and Unified Parkinson's Disease Rating Scale (UPDRS) [14,19-26,30].

Quality of life was assessed in three studies: one used the 39-item Parkinson's Disease Questionnaire (PDQ-39) [19], another its short form (PDQ-8) [26] and two studies used the 36-item Short Form Health Survey (SF-36) [20,26].

The cognitive status of the participants was assessed in most studies with the Mini Mental State Examination. Demented patients were excluded from eight studies [14,19,21-26].

In most studies adverse effects were reported spontaneously and verbally to the investigators or via questionnaires. In three studies the authors used the "Udvalg for Kliniske Undersogelser" side effects rating scale (UKU) [31,21,24,25]. All drug treatments were generally well tolerated. Common side effects for the SSRIs group included nausea, fatigue/asthenia and diarrhoea and for the TCA group dry mouth, somnolence, constipation and orthostatic hypotension.

Overall 192 patients were randomised to an SSRI (65 to fluoxetine, 56 to sertraline, 33 to citalopram, 20 to fluvoxamine and 18 to paroxetine), and 238 to a comparator group (58 patients to placebo, 83 patients to amitriptyline, 17 to desipramine, 17 to nortriptyline and 64 to other treatments including pramipexole, nefazodone and rTMS). In total 430 patients were enrolled in the studies. There were 343 patients who completed the studies (overall completion rate 80%). A detailed narrative description of the studies, including assessment of methodological quality and reporting of common side-effects, is given in additional file 2.

### Meta-analysis results

#### a) Comparison 1: SSRIs versus Placebo

Four trials had a separate placebo arm: two citalopram studies [21,22], one sertraline study [14] and one paroxetine study [26]. The Wermuth et al. study [21] did not report the dichotomous response but we obtained the data after contacting the authors (Dr Wermuth).

Since this comparison was the main aim of the analysis, in order to use all possible available evidence we also considered the inclusion in the meta-analysis of the following two studies:

a) the study by Antonini et al. [19] compared standard dose sertraline to a very low dose of amitriptyline (25 mg/day). This dose is not normally considered as having antidepressant potency and a meta-analysis of the efficacy of low versus standard dose amitriptyline generally identified papers with doses not less than 37.5 mg/day [32]. Therefore one can consider this dose as an active placebo with the added advantage of a diminished unblinding effect [33]. Inclusion of this sertraline trial in the analysis would result in the increase of the number of patients randomized to sertraline from the 6 included in the Leentjens et al. study [14] to 22 as 16 patients in the Antonini et al. study [19] were randomized to this treatment.

b) the study by Fregni et al. [25] compared fluoxetine plus sham rTMS with placebo plus rTMS (total N = 43). Given that rTMS is still considered as experimental in the treatment of depression and a systematic review found insufficient evidence to support its use in depression [34], we also considered this study as a predominantly SSRI vs placebo comparison. In any case we have repeated the analyses after excluding the two additional studies for comparison.

**Figure 2:**
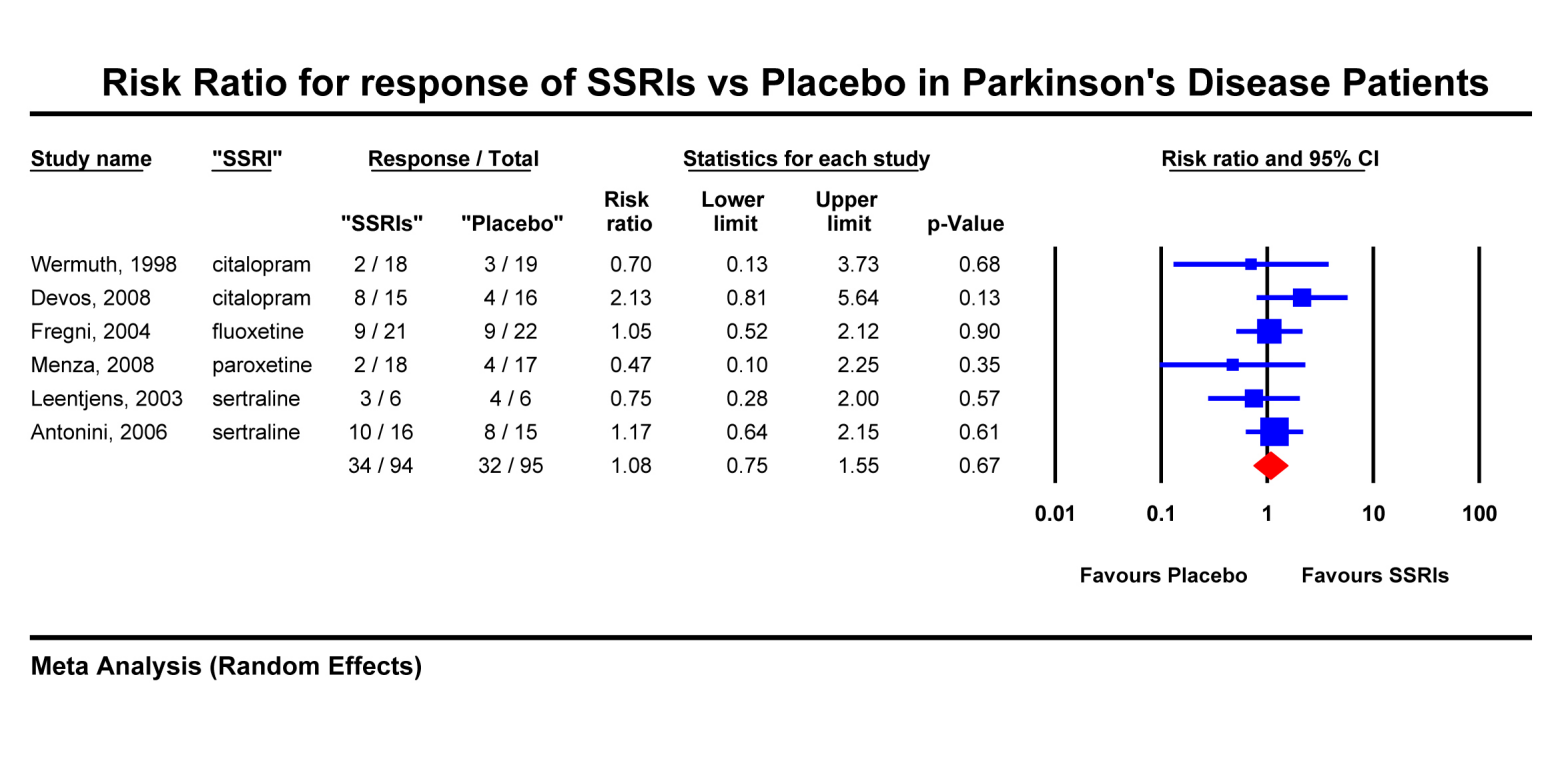
**Risk Ratio for response of SSRIs vs Placebo in Parkinson's Disease Patients**.

Treatment response was assessed using the HDRS in four studies [19,21,25,26] and the MADRS in two studies [14,22]. Figure 2 shows the results of our full analysis with six studies and 189 patients included. The observed response rate for SSRIs was 36% and that of placebo 34%. The combined risk ratio (random effects) was 1.08 and the 95% confidence interval was 0.75 to 1.55 (p = 0.67). Statistical tests did not show significant heterogeneity (Q = 3.82 with 5 degrees of freedom, p = 0.57). Exploration of the forest plot shows that one study, Devos et al. [22], is the only study with a trend for a favorable response for SSRIs (citalopram) with a risk ratio of 2.13 and 95% CI 0.81 - 5.64 (p = 0.13). Pooling, however, the two available citalopram studies [21,22] reduced the risk ratio for citalopram to 1.52 (95% CI 0.55 - 4.17, p = 0.42). Excluding the Antonini et al. [19] and Fregni et al. [25] studies, the results were not different with the combined risk ratio closer to unity than before (risk ratio = 0.99, 95% CI of 0.51 to 1.95, p = 0.98).

**Figure 3:**
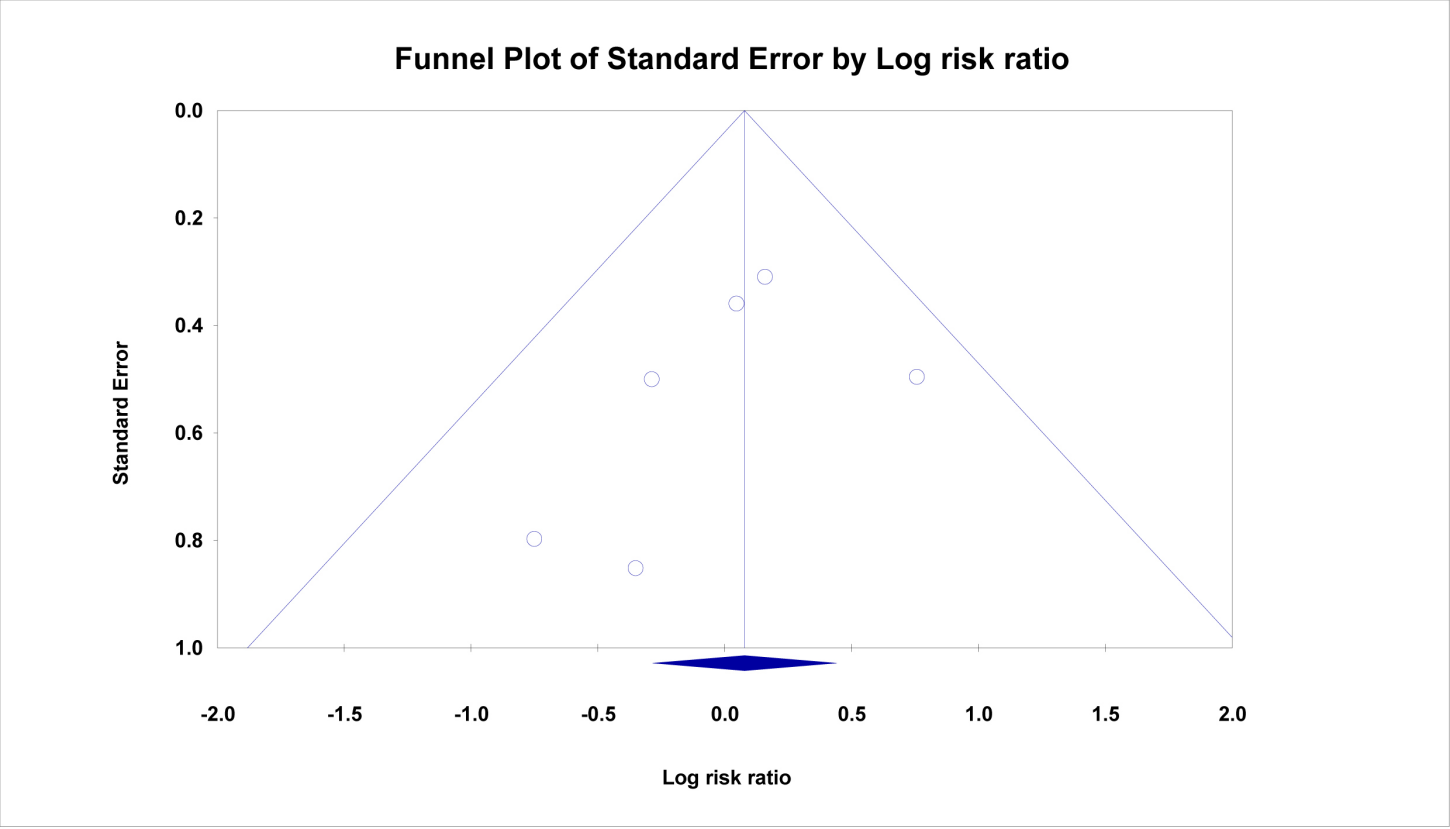
**Publication Bias in SSRIs vs Placebo trials of depression in PD**.

A funnel plot is presented in figure 3. It can be seen that small trials with negative results have been published but small trials with positive results are missing. Publication bias is more likely when small negative trials are missing and this is not the case here. It should be noted, however, that for a more accurate assessment of publication bias a larger number of studies is usually needed.

Since the results were negative we also tested whether the use of the continuous outcome (standardized mean differences of the endpoint scores on the HDRS or MADRS) would make any difference compared to the dichotomous response. One study [22] was excluded from this analysis because due to non-normal data the authors reported medians and quartiles only. For the remaining five studies, the standardized mean difference using the Hedges' g estimate was -0.13 (95% CI: -0.43 - 0.17, p = 0.40) with no evidence of heterogeneity (Q = 2.71 with 4 degrees of freedom, p = 0.61). Details of this analysis and a forest plot are provided in additional file 3.

#### b) Comparison 2: SSRIs versus TCAs

Five studies had used an older TCA as the comparator: three amitriptyline studies [19,23,27] one desipramine study [22] and one nortriptyline study [26]. As explained in the previous section the Antonini et al. study [19] used a very low dose of amitriptyline and was excluded from this analysis. Moreover, the Serrano-Duenas study [23] had several methodological limitations with high risk of bias which made its inclusion problematic (no reporting of binary outcome, CONSORT guidelines were not followed, there is no flow chart of the randomization process, there is no information on eligibility criteria, potential unblinding problems since the SSRI was given in the morning and amitriptyline at night). Since the study did not report a binary outcome we decided not to include it in the meta-analysis. Therefore, for this analysis we included three studies. It should be noted however, that one of the studies [27] is only in abstract form and was never published as a full paper.

**Figure 4:**
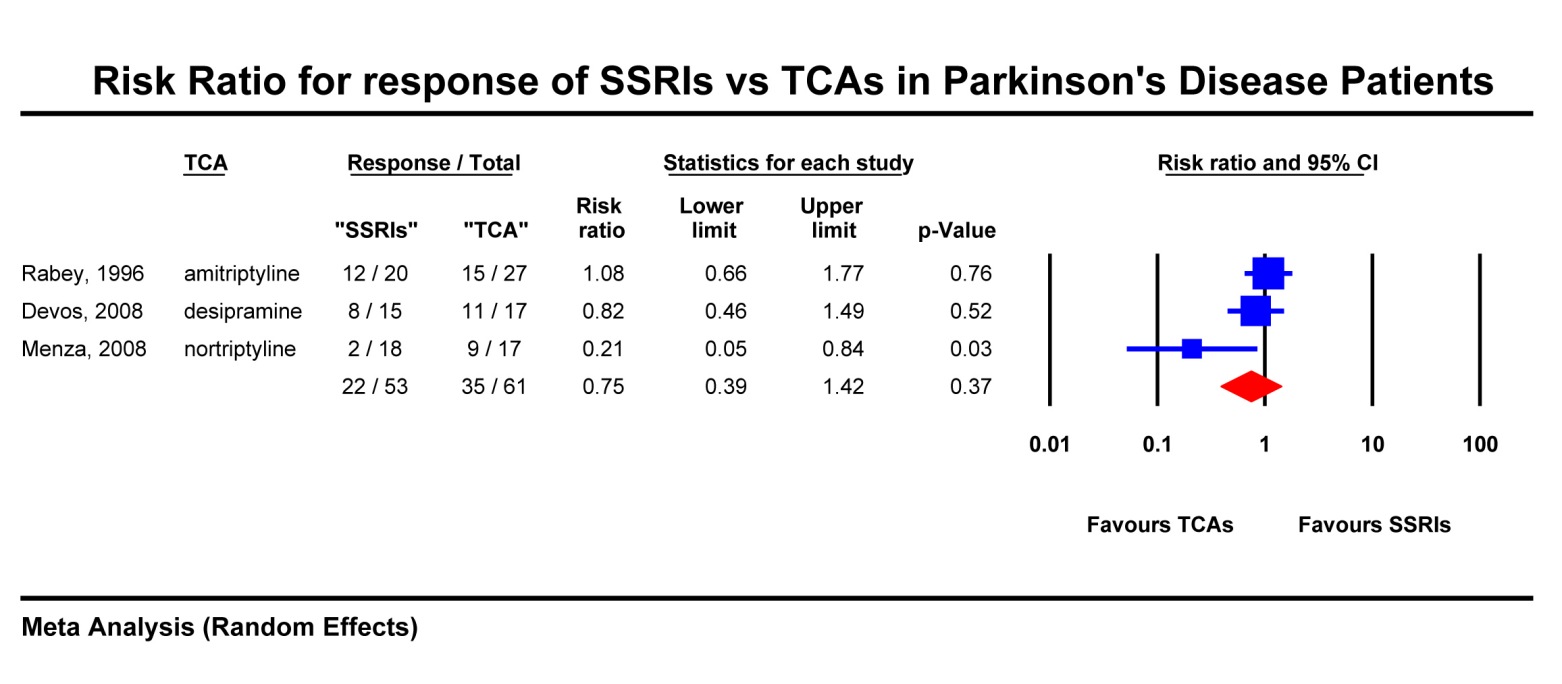
**Risk Ratio for response of SSRIs vs TCAs in Parkinson's Disease Patients**.

Treatment response was assessed using the HDRS in two studies [26,27] and the MADRS in one study [22]. Figure 4 shows the results of this analysis with three studies included (total N = 114). The observed response rate for SSRIs was 41% and that of TCAs 57%. The combined risk ratio (random effects) was 0.75 with the 95% CI 0.39 - 1.42 (p = 0.37).

#### c) Acceptability Analysis

**Figure 5:**
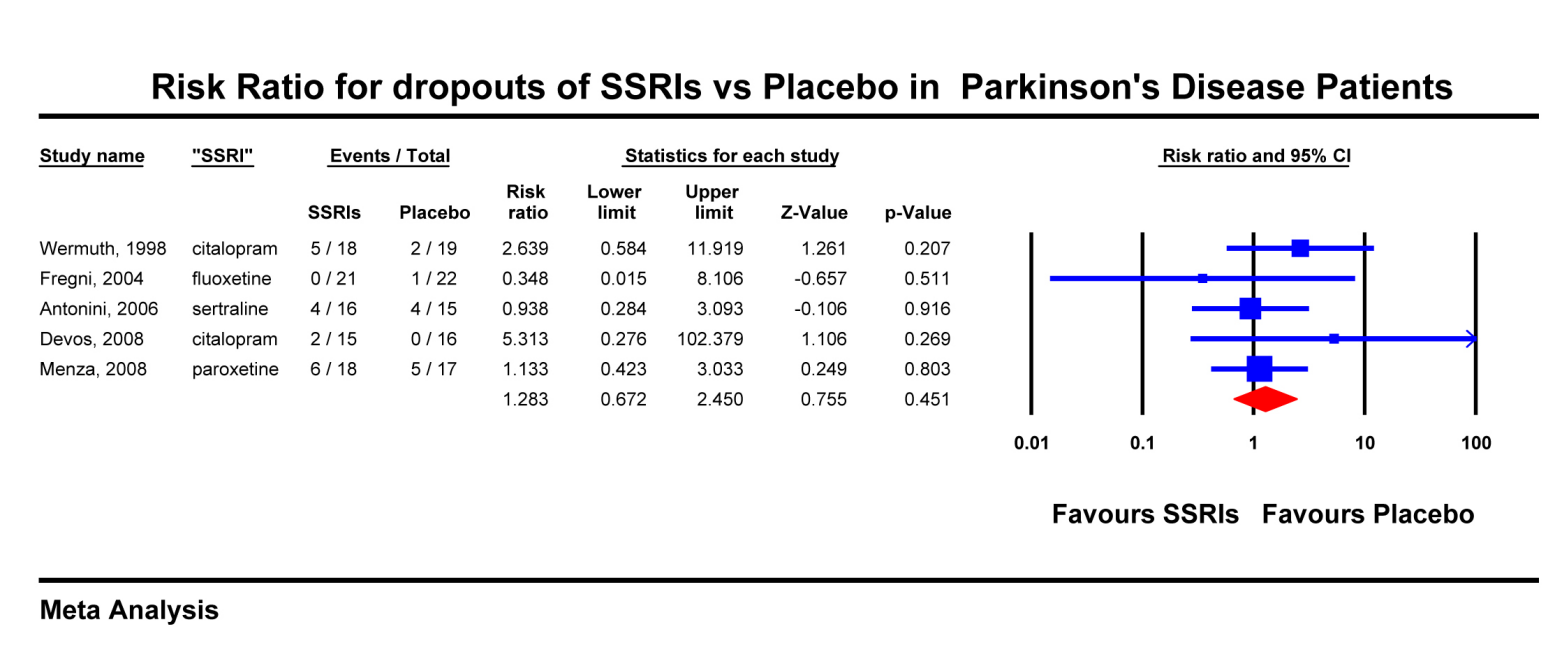
**Risk Ratio for dropouts of SSRIs vs Placebo in Parkinson's Disease Patients**.

We first compared total dropouts in SSRIs vs. placebo. Five studies were included in this analysis as one small study had no dropouts. 17 out of 88 patients on SSRI dropped out (19.3%) vs. 12 out of 89 on placebo (13.5%). The combined risk ratio for dropouts was 1.28 with a 95% CI of 0.67 - 2.45 (figure 5). It should be noted that citalopram showed a greater tendency for increased dropout rates but this result was based on only two studies (combined risk ratio for citalopram 3.05, 95% CI 0.80 - 11.68, p = 0.10).

In the three studies that compared SSRIs with TCAs and were included in the efficacy analysis, the dropouts did not differ between the two classes of drugs (30% for SSRIs vs 31% for TCAs). The combined risk ratio was 0.96 with a 95% CI of 0.56 - 1.64 (p = 0.88).

A more detailed description of the side effects reported in each study is given in the additional file 2.

## Discussion

### Main Findings

In the present meta-analysis there was insufficient evidence to reject the null hypothesis of no treatment differences between SSRIs and placebo in the treatment of depression in the context of PD. The crude response rate in SSRIs was 36% versus 34% in placebo and the combined risk ratio (random effects) was 1.08 (95% CI 0.77 - 1.55). The comparison of SSRIs with TCAs was based on only three studies, one of them only published in abstract form, and the risk ratio was 0.75 (0.39 - 1.42) with a crude response rate of 41% vs. 57%.

### Limitations

Several limitations of this study should be considered. First, this analysis included a limited number of small studies and therefore type-II errors (i.e. false negative results) due to chance cannot be entirely excluded as an alternative explanation for our main finding. This issue is even more important regarding specific antidepressants. Therefore we cannot exclude the possibility that specific SSRIs may in the future show evidence of superiority compared to placebo. Second, patients included in the trials are not always representative of the patients seen in real clinical practice. Most trials excluded patients with dementia; severe motor fluctuations; comorbid medical disorders; and symptoms of psychotic depression. Therefore, our results should not be generalized in such patients. Third, we selected to use as our primary outcome the (dichotomous) antidepressant response and not the continuous outcomes (standardized mean differences at endpoints). We did that on the basis that the concept of response is useful to investigate the significance of the results both from a statistical and a clinical point of view. Antidepressant response has been extensively used as the primary end point for defining improvement in many trials [35,36]. The use of continuous measures has been criticized because of its inability to discriminate between an effect that is clinically insignificant versus an effect that is clinically significant [37-39]. Other studies have used an arbitrary three-point difference in the Hamilton Rating Scale for Depression (equivalent to a standardized mean difference of 0.5) as indicative of a clinically significant change [40]. The few empirical studies, however, do not support this threshold and point to larger effects that are often observed in response rates [37]. We would like to note however, that in our main comparison (SSRIs versus placebo) we also carried out an analysis with the continuous outcome and the result was not different. Fourth, studies were generally small and most of them had a total sample of less than 50 patients. Although in theory randomization eliminates the problem of unknown confounding factors, an imbalance in the two arms cannot be excluded given the small number of patients randomized. These problems are more likely in the comparison between SSRIs and TCAs as the total number of trials included was smaller (three). Finally, in our main comparison, we selected to include two studies that were not designed as typical placebo-controlled trials because we considered that their inclusion could be justified for the reasons we mentioned in the relevant section and in order to minimize the possibility of type II errors. In any case, exclusion of these studies did not alter our results.

### Interpretation of the Results

Due to the small number of studies and the small sample sizes we cannot exclude the possibility that the reported lack of efficacy of SSRIs compared to placebo is a type II error (i.e. a false negative result). If, however, there is a true lack of effect what are the possible explanations? First, it would be informative to compare the response rates we found in our analysis with those reported from general depression trials as the lack of efficacy could be due to a low response rate of both SSRIs and placebo, indicating a general lack of response in trials of depression in the context of PD. A meta-analysis that investigated time trends of the placebo response rates in antidepressant trials [41] reported an average response rate for SSRIs of 48.9% (standard deviation: 10.3) while the corresponding figure for placebo was 30% (standard deviation: 8). Therefore, it seems that the lack of efficacy of SSRIs in our analysis is probably due to a lower than expected response rate for SSRIs (36% in our analysis) while the response rates we found for placebo (34%) were very close to those reported in the general depression literature. This observation does not support the view that there is a general lack of response in depression trials of PD patients, but rather that SSRIs fail to achieve a response comparable to the one achieved in general depression trials. Second, the findings could be explained by measurement bias. It is known that assessing the diagnostic criteria and severity of depression in PD patients is a difficult task due to several overlapping symptoms (psychomotor changes, apathy, fatigue, insomnia, sleep disorders, weight loss, cognitive dysfunction, social withdrawal) [9,42]. It has been suggested that the depression diagnostic criteria should be modified to assess depression more accurately in PD patients [43]. In addition, assessment of depressive symptoms in the context of PD is usually done with a non-etiological and "inclusive" approach, where the evaluator assesses all symptoms of depression irrespectively of the possible etiology and whether some of the symptoms are secondary to PD [43]. However, if these measurement issues were the reason behind the relative lack of efficacy of SSRIs one would also expect a lower placebo response compared to general depression trials, and this was not found as explained before. Third, selection bias could also explain our results. RCTs usually exclude patients with the more severe depression, but these patients are more likely to respond to SSRIs [40]. Most trials have excluded subjects with suicidal ideation or psychotic depression. In addition, most of the patients included in the trials had moderate levels of depression although there were exceptions [22,25]. Therefore we do not know whether inclusion of patients with severe or very severe levels of depression could influence the results in favor of the SSRIs. In any case the results of the present analysis should not be generalized beyond the level of moderate severity of depression. Fourth, there is the possibility that treatment of depression in the context of PD may require higher doses of antidepressants compared to non-comorbid depression. Most studies included in the analysis have used typical doses and it is not known whether an increase in dose (or duration of treatment) could improve the response rates of SSRIs.

Further support for a relative inefficiency of SSRIs in the treatment of depression comorbid with PD is coming from neurobiological studies. There is strong evidence that serotonin dysfunction plays a key role in the pathophysiology of major depression [44]. However, this refers to depression not comorbid with PD. Research that has been specifically carried out in depressed patients with PD does not support the serotonergic dysfunction in this subgroup. A recent review of imaging studies in PD concluded that there is very little evidence to support a major role for serotonergic system in regulating depression in the context of PD [45]. In contrast there is strong evidence for the role of both the noradrenergic [45] and dopaminergic systems [46].

## Conclusion

SSRIs are prescribed in depression more often than any other class of antidepressants and this is also true for depression in the context of PD [7]. In the U.S. survey of the treatment of depression in PD [7], 63% of the antidepressant prescriptions were for SSRIs and 7% only for TCA. Similar trends are expected in other countries. Our results show that the current clinical practice is not supported by strong randomized evidence. There is still uncertainty on the efficacy of SSRIs in depression in the context of PD and clinicians should be aware of this uncertainty. Based upon the results of our analysis we cannot exclude the possibility that SSRIs in the future may show evidence of effectiveness, especially for severe or very severe depression. The small number of studies does not also permit us to recommend TCAs routinely and more placebo-controlled trials are needed. In addition it is not known whether tertiary amine TCAs that act on both serotonin and noradrenaline are better than secondary amine TCAs that act predominantly on noradrenaline. We should note however, that in two of the three studies that we included in our SSRIs versus TCAs comparison the investigators preferred to use secondary amines [22,26]. Given this uncertainty clinicians could consider using secondary amine TCAs at least as often as SSRIs.

There are implications for future research as well. The role of other antidepressant drugs should be further investigated, particularly those that act on noradrenaline or dopamine. We know that a major randomized trial of venlafaxine, a dual re-uptake inhibitor (SNRI) is currently conducted in the US, comparing this drug with paroxetine and placebo, but results are not expected before 2011 (see: http://www.clinicaltrials.gov/ct2/show/NCT00086190). The role of other potentially interesting drugs should also be explored. Mirtazapine, enhances both serotonergic and adrenergic neurotransmission [47] and a case series reported that it improved parkisonian tremor [48]. No studies have been carried out however for its use as an antidepressant in PD. Bupropion, is also a licensed antidepressant which acts as a dopamine reuptake inhibitor and its role in treating depression in PD could be explored further. Finally, future studies should have a more pragmatic design and include patients with more severe depression (including those with suicidal ideation or psychotic symptoms) as this is a group of patients that is commonly seen in clinical practice and could benefit more from antidepressant treatment.

## Abbreviations

BDI: Beck Depression Inventory; HDRS: Hamilton Depression Rating Scale; MADRS: Montgomery-Asberg Depression Rating Scale; PD: Parkinson's Disease; r-TMS: repetitive transcranial magnetic stimulation; SSRI: Selective Serotonin Reuptake Inhibitors; TCA: Tricyclic antidepressants; UPDRS: Unified Parkinson's Disease Rating Scale.

## Competing interests

Authors PS, GL and VM have served as speakers and have received speaking fees from pharmaceutical companies that manufacture and distribute SSRIs. All other authors declare no conflict of interest in relation to this paper.

## Authors' contributions

PS was responsible for the conception of the study, was the principal writer of the paper and contributed to the study design, data extraction, analysis and interpretation of the findings. EB contributed to data extraction and preparation of the paper. GS contributed to the statistical analysis and critical interpretation of the findings. GL contributed to the analysis, writing and critical interpretation of the findings. AK contributed to the writing and critical interpretation of the findings. VM contributed to the analysis, interpretation and writing of the paper. All authors read and approved the final manuscript.

## Pre-publication history

The pre-publication history for this paper can be accessed here:

http://www.biomedcentral.com/1471-2377/10/49/prepub

## Supplementary Material

Additional file 1 **Table s1: Table of excluded studies**. Click here for file

Additional file 2 **Detailed narrative description of the included studies**. Click here for file

Additional file 3 **Details of the comparison between SSRIs and placebo using the continuous outcome**. Click here for file
