# Supplementary material for: Efficacy and acceptability of selective serotonin reuptake inhibitors for the treatment of depression in Parkinson's disease: a systematic review and meta-analysis of randomized controlled trials
Source: BMC Neurol. 2010 Jun 21;10:49. doi: 10.1186/1471-2377-10-49 (PMC2903535; doi:10.1186/1471-2377-10-49)
Supplement: Additional file 1 — Table s1: Table of excluded studies. [file 1471-2377-10-49-S1.DOC]

**Additional File 1**

Paper: Skapinakis et al. Efficacy and acceptability of selective serotonin reuptake inhibitors for the treatment of depression in Parkinson’s Disease: Systematic review and meta-analysis of randomized controlled trials.

**Table s1: Table of Excluded Studies (N=14)**

|  | **Study** | **Reason for Exclusion** |
| --- | --- | --- |
| **1** | Arsland D, Larsen JP, Lim NG, Wermuth L, Bech P. α_2_-Adrenoreceptor antagonism and serotonin reuptake inhibition in patients with Parkinson disease and depression. Nord J Psychiatry 2000;54(6):411-415. | a |
| **2** | Ceravolo R, Nuti A, Piccinni A, Dell’Agnello G, Bellini G, Gambaccini G, Dell’Osso L, Murri L, Bonuccelli U. Paroxetine in Parkinson’s disease: Effects on motor and depressive symptoms. Neurology 2000;55(8):1216-1218. | a |
| **3** | Dell’Agnello, Ceravolo R, Nuti A, Bellini G, Piccinni A, D’Avino C, Dell’Osso L, Bonuccelli U. SSRIs do not worsen Parkinson’s disease: evidence from an open-label, prospective study. Clin Neuropharmacol. 2001;24(4):221-227. | a |
| **4** | Hauser RA, Zesiewicz TA. Sertraline for the treatment of depression in Parkinson’s disease. Mov Disord. 1997:12(5):756-759. | a |
| **5** | Kulisevsky J, Pagonabarraga J, Pascual-Sedano B, Gironell A, Garcia-Sanchez C, Martinez-Corral M. Motor changes during sertraline treatment in depressed patients with Parkinson’s disease. Europ J Neurol 2008;15:953-959. | a |
| **6** | Marino S, Sessa E, Di Lorenzo G, Digangi G, Alagna A, Bramanti P, Di Bella P. Sertraline in the treatment of depressive disorders in patients with Parkinson’s disease. Neurol Sci. 2008;29(6):391-395. | a |
| **7** | Meara RJ, Howmick BK, Hobson JP. An open uncontrolled study of the use of sertraline in the treatment of depression in Parkinson’s disease. J Serotonin Research 1996;4:243-249. | a |
| **8** | Menza M, Marin H, Kaufman K, Mark M, Lauritano M. Citalopram treatment of depression in Parkinson’s disease: the impact on anxiety, disability and cognition. J Neuropsychiatry Clin Neurosci 2004;16(3):315-319. | a |
| **9** | Montastruc JL, Fabre N, Blin O, Senard JM, Rascol O, Rascol A. Does fluoxetine aggravate Parkinson’s disease? A pilot prospective study. Mov Disord. 1995;10(3):355-357. | a |
| **10** | Rampello L, Chiechio S, Raffaele R, Vecchio I, Nicoletti F. The SSRI citalopram improves bradykinesia in patients with Parkinson’s disease treated with L-dopa. Clin Neuropharmacology 2002;25(1):21-24. | a, b |
| **11** | Rihmer Z, Satori M, Pestality P. Selegiline-citalopram combination in patients with Parkinson’s disease and major depression. Int J Psych Clin Pract 2000;4:123-125. | a |
| **12** | Tesei S, Antonini A, Canesi M, Zecchinelli A, Mariani CB, Pezzoli G. Tolerability of paroxetine in Parkinson’s disease: a prospective study. Mov Disord. 2000;15(5):986-989. | a |
| **13** | Weintraub D, Taraborelli D, Morales KH, Duda JE, Katz IR, Stern MB. Escitalopram for major depression in Parkinson’s disease: an open label, flexible-dosage study. J Neuropsychiatry Clin Neurosci. 2006;18(3):377-383. | a |
| **14** | Wittgens W, Donath O, Trenckmann U. Treatment of depressive syndromes in Parkinson’s disease (PD) with paroxetine. Mov Disord. 1997;12(suppl 1):128. | a |
| ^a^ uncontrolled study | | |
| ^b^ non-depressed patients included | | |
