# Supplementary material for: Efficacy and acceptability of selective serotonin reuptake inhibitors for the treatment of depression in Parkinson's disease: a systematic review and meta-analysis of randomized controlled trials
Source: BMC Neurol. 2010 Jun 21;10:49. doi: 10.1186/1471-2377-10-49 (PMC2903535; doi:10.1186/1471-2377-10-49)
Supplement: Additional file 2 — Detailed narrative description of the included studies. [file 1471-2377-10-49-S2.DOC]

**Additional File 2**

Paper: Skapinakis et al. Efficacy and acceptability of selective serotonin reuptake inhibitors for the treatment of depression in Parkinson’s Disease: Systematic review and meta-analysis of randomized controlled trials.

**Detailed Description of the included studies**

**Comparison 01: SSRI vs. placebo.**

Four studies compared the efficacy of SSRIs and placebo in the treatment of depression in Parkinson’s disease. Two of them (Devos et al., 2008 and Menza et al., 2008) also had a third TCA arm and are mentioned in the next section as well.

1. *Wermuth et al., 1998. (21)*

Study description: Wermuth et al. (1998) conducted a randomized placebo-controlled trial in 37 patients with idiopathic Parkinson’s disease in order to assess the efficacy of citalopram as an antidepressant agent. Major depression was diagnosed according to the DSM-III-R. Demented patients were excluded from the trial. The trial had an acute phase (6 weeks duration) and an open-label continuation phase (46 weeks). The dose of citalopram depended on the age and clinical response. In the acute phase patients in the citalopram group younger than 65 years received 20mg/day citalopram and those older than 65 years received 10mg/day. At the end of the acute phase the dose of citalopram was doubled for patients who had not responded. The main assessment tools used by the authors were HDRS, MES and UPDRS. The primary outcome of the study was the change in HDRS scores from baseline at the end of the acute phase. Regarding the methodological quality, the study did not describe the method used to conceal the allocation sequence. In addition, the study is described as double blind without further information.

Efficacy: The authors found a significant treatment effect in both groups but they were unable to show any between-group differences. Response was 11% (2 out of 18) for the citalopram group and 16% (3 out of 19) for the placebo group.

Safety: The authors used the UKU scale to record the side effects in the acute phase of treatment. Overall citalopram was well tolerated. Increased sweating (22% vs. 5%), nausea and vomiting (17% vs. 16%), diminished sexual desire (11% vs. 5%) and diarrhoea (6% vs. 0%) were more common in the citalopram-treated patients compared to the placebo group. Five patients (28%) in the citalopram group discontinued the treatment vs. two patients in the placebo group (11%).

*b) Leentjens et al., 2003. (14)*

Study description: Leentjens et al. (2003) conducted a double bind randomized controlled trial of sertraline vs. placebo in the treatment of depression in Parkinson’s disease. 12 patients with Parkinson’ disease according to the UK-PDS-BB and major depression according to DSM-IV criteria with a mean age of 67±7.8 years were included in the study (6 in each group). The authors reported that recruitment of patients was very difficult and although they aimed to recruit 40 patients in 30 months only 12 patients were finally entered into the study. All patients had a high score on the Mini Mental State Examination (mean MMSE was 27.8 with an sd of 2.3). The duration of the study was 10 weeks. The sertraline group started with a dose of 25mg, which was increased to 50mg after 1 week. If there was no response after 6weeks of treatment the dose of sertraline was doubled to 100mg. The primary outcome measure was the reduction of the score on the MADRS. A 50% reduction of the pre-treatment MADRS score was defined as a response. Regarding methodological quality, the study had a low risk of bias but the small sample size is a limiting factor.

Efficacy: There was no statistically significant difference between the two groups. Three out of 6 patients in the sertraline group responded (50%) versus 4 out of 6 in the placebo group (67%).

Safety: No adverse effects or dropouts are reported by the authors.

*c) Devos et al., 2008 (22)*

Study description: Devos et al. (2008) studied the efficacy of citalopram as an antidepressant agent in 48 patients with PD by conducting a randomized double blind placebo controlled study with three parallel arms. One arm of the study received citalopram (20mg/day), the second desipramine (75mg/day) and the third placebo. The study duration was 4 weeks. Participants were between 56 and 68 years. Major depression was diagnosed according to the DSM-IV criteria. Demented patients were excluded. Depression was assessed with MADRS. The primary endpoint was the change in the MADRS score after 2 and 4 weeks of treatment. The authors also evaluated the response in each group which was defined as an at least 50% reduction of baseline MADRS score. This study was of high methodological quality with a low risk of bias.

Efficacy: Data extraction for calculating response was made from Table 2 of the paper as we noted an inconsistency on the total number of patients between the flow chart of the study and table 2. After 4 weeks of treatment citalopram significantly improved major depression compared with placebo. Out of 15 patients treated with citalopram 8 responded to treatment (53%) versus 4 out of 16 patients in the placebo group (25%).

Safety: In total citalopram was well tolerated. Severe adverse effects occurred in two citalopram treated patients (13%) and included worsened bradykinesia and induction of erectile dysfunction. All severe adverse effects resolved after discontinuation. In the placebo group there were no dropouts.

*d) Menza et al., 2008 (26)*

Study description: Menza et al. (2008) conducted a randomized double-blind placebo controlled trial with 52 patients with PD and depression (mean age 62.8 years). There were three parallel arms in the study. One group received controlled release paroxetine (paroxetine CR) (mean dose: 28.4mg), the second group received placebo and the third group received nortriptyline (mean dose: 48.5mg). Demented patients were excluded. The study duration was 8 weeks.. There were two primary endpoints set by the authors: the decrease in the HDRS scores and the percentage of depression responders in each treatment group. This study had a high methological quality with a low risk of bias.

Efficacy: Paroxetine CR was not found superior to placebo. At the end of the treatment 2 out of 18 patients receiving paroxetine CR responded (11%) versus 4 out of 17 patients in the placebo group (24%).

Safety: Paroxetine CR was generally well tolerated although a significantly higher average number of side effects in this treatment group was recorded compared to placebo. Fatigue (17%) and dry mouth (6%) were more common in the paroxetine group comapred to the placebo group (12% and 0% respectively). Seven out of 18 patients (39%) discontinued the treatment in the paroxetine group vs. 6 out of 17 in the placebo group (35%).

**Comparison 02: SSRI vs. TCA**

We included five studies comparing the therapeutic effect of an SSRI with a tricyclic antidepressant (TCA) in depression in the context of PD.

*a) Rabey et al., 1996 (27)*

Study description: Data for this trial were extracted from the Cochrane review (Ghazi-Noori et al., 2005). Rabey et al. (1996) conducted a randomized controlled trial to compare fluvoxamine with amitriptyline in 47 PD patients with a mean age of 75 years. Depression was diagnosed according to the DSM-IV criteria. The authors reported that 17 of the patients had dementia. Fluvoxamine was given at a mean dose of 78mg/day and amitriptyline was given at a mean dose of 69mg/day. Study duration was 16 weeks. The primary outcome measure was response defined as an at least 50% reduction of baseline HDRS scores. This study had a high risk of bias since it was unblinded and the authors did not describe the method they used for the random allocation of the patients. In addition, this trial was only published in abstract form and not in a full paper.

Efficacy: 12 out of 20 (60%) participants treated with fluvoxamine showed a 50% decrease in HDRS score versus 15 out of 27 (56%) in the amitriptyline group. There was no statistical difference between the two groups.

Safety: Eight patients treated with fluvoxamine (40%) and 12 treated with amitriptyline (44%) dropped out. This was due to confusion and visual hallucinations in 7 fluvoxamine (35%) vs. 10 (37%) amitriptyline patients and tremor in one fluvoxamine patient (5%) vs. none in the amitriptyline group. Dryness of the mouth (4%) and somnolence (4%) were reported only in the amitriptyline group

*b) Serrano-Duenas, 2002 (23)*

Study description: Serrano-Duenas (2002) compared the antidepressant effect of fluoxetine with that of amitriptyline in a randomized controlled study which included 77 patients with a mean age of 68.2 years. The study duration was 12 months. The first evaluation of the patients occurred after three months of treatment. 37 patients received fluoxetine (mean dose 27.3mg/day) and 40 patients received amitriptyline (mean dose 35.2mg/day). Demented patients were excluded (mean MMSE scores were 33.4 with an sd of 2.4). The author used the HDRS to assess the severity of depression. The primary outcome was change in the HDRS score from baseline. The study had a high risk of bias, since it was unblinded and the author did not describe the method he used for the random allocation of subjects. This study had additional limitations (no reporting of binary outcome, consort guidelines were not followed, there is no flow chart of the randomization process, there is no information on eligibility criteria, potential unblinding problems since the SSRI was given in the morning and amitriptyline at night) that prevented us from using it in the quantitative synthesis.

Efficacy: The patients in the amitriptyline group showed a significant improvement in HDRS score compared with those treated with fluoxetine after three months of treatment.

Safety: There were 7 dropouts in the fluoxetine group (19%) vs. 12 dropouts in the amitriptyline group (30%). Overall fluoxetine was well tolerated since the author didn’t mention any side effects in this group. Side effects in the amitriptyline treated group included dry mouth (7,5%), somnolence (5%), orthostatic hypotension and instability (2.5%).

*c) Antonini et al., 2006 (19)*

Study description: Antonini et al. (2006) conducted a randomized controlled trial to compare the effect of sertraline (50mg) versus low dose amitriptyline (25mg) on depression in 31 patients with Parkinson’s disease. The study duration was 12 weeks.. The patients fulfilled the diagnostic criteria for PD according to the UK-PDS-BB and were diagnosed with major depression according to the DSM-IV criteria. Demented patients were excluded. The primary endpoints set by the investigators were the reduction in HDRS scores and the response rate defined typically as in other studies in each group. Regarding methodological quality, potential problems for this study were that it was single blinded and the authors did not describe the method they used for the random allocation of subjects.

Efficacy: The authors reported a significant reduction in HDRS scores for both groups combined but they did not report the between group difference because as they pointed out: “We did not seek a direct comparison of the antidepressant efficacy between the two drugs because the amitriptyline dose we were allowed to use, was at the lowest end of the range for geriatric depression and not comparable with sertraline”. Regarding response, 10 out of 16 patients treated with sertraline responded (63%) versus 8 out of 15 in the low dose amitriptyline group (53%). We should like to note that based on the authors’ views and our own interpetation of the literature we considered this trial as a predominantly SSRI versus placebo comparison since the low dose amitriptyline can be considered as an active placebo in this trial.

Safety: The authors reported that adverse effects occurred in four patients (25%) in the sertraline group, which were nausea (12,5%), confusion (6%), hypotension (6%) and led them to discontinuation of the study. In the amitriptyline group four patients (27%) also discontinued the study due to confusion and visual hallucinations (13%), sleepiness (7%) headache and tachycardia (7%).

*d) Devos et al., 2008 (22)*

Study description: Details are given in the previous section.

Efficacy: After 4 weeks of treatment no significant difference between citalopram and desipramine group were reported. Out of 15 patients treated with citalopram 8 responded to the treatment (53%) versus 11 out of 17 patients in the desipramine group (65%).

Safety: Citalopram was better tolerated than desipramine. Mild adverse effects such as dry mouth, constipation, nausea, headache and hyperhidrosis were twice as frequent in the desipramine group as in the citalopram group. One desipramine treated patient discontinued due to orthostatic hypotension worsening (6%).

*e) Menza et al., 2008 (26)*

Study description: Details are given in the previous section.

Efficacy: The nortriptyline response rate was significantly higher compared with the paroxetine CR group. Nine out of 17 nortriptyline treated patients responded to the treatment (53%) versus 2 out of 18 paroxetine CR treated patients (11%).

Safety: The nortriptyline group had more anticholinergic side effects including constipation (35%) and dry mouth (41%) compared to paroxetine CR (6% and 6% respectively). Five out of 17 patients in the nortriptyline group dropped out (29%) vs. seven out of 18 patients in the paroxetine CR group (39%).

**Comparison 03: SSRI vs. other treatments**

Three studies were included in this section.

*a) Avila et al., 2003 (24)*

Study description: Avila et al. (2003) compared the efficacy and safety of fluoxetine versus nefazodone in the treatment of depression in PD patients in a single blind randomised trial. Sixteen patients with Parkinson’s disease and major depression or dysthymic disorder diagnosed according to the DSM-IV criteria were randomised into the two groups. The first group (n=7) received fluoxetine (mean dose 25mg/day) and the second group (n=9) received nefazodone (mean dose 200mg/day). The mean age was 70.4 (sd=6.4) years. Demented patients were excluded. The study duration was 90 days. The primary outcome was the reduction from baseline in the BDI scores between the two groups. The UKU rating scale was used for the evaluation of the side effects. Regarding methodological quality, the study was single blinded and assessment of depression could have been influenced by the lack of blinding.

Efficacy: There was a significant improvement in depressive scores compared to the baseline for both groups but there were no between group differences (p=0.97).

Safety: Mild adverse effects occurred in both groups and included asthenia, anxiety, orthostatic dizziness and constipation. Three of the nefazodone-treated patients (33%) discontinued the study because of increased tremor (2) and diarrhoea (1).

*b) Fregni et al., 2004 (25)*

Study description: Fregni et al. (2004) compared fluoxetine (20mg) with repetitive transcranial magnetic stimulation (rTMS) in 43 depressed PD patients in a double blind randomized trial. The patients were diagnosed with depression according to the DSM-IV criteria. Demented patients were excluded. The study duration was 8 weeks.. The first group (21 patients) received 20mg/day fluoxetine and sham rTMS and the second group (22 patients) received placebo and active rTMS (15Hz for ten days). The primary outcome was response typically defined as in other studies using the HDRS. Regarding methodological quality, this study had a low risk of bias.

Efficacy: No differences were recorded between the two groups. In the fluoxetine group 9 out of 21 patients responded (43%) versus 9 out of the 22 patients in the active rTMS group (41%). As we explain in the main text, given that rTMS is not yet considered a standard treatment for depression we considered this study as a predominantly SSRI vs placebo comparison and we have included it in our primary comparison to increase the sample size and the power of the analysis.

Safety: The authors reported that adverse effects were more common in the fluoxetine group, but they didn’t name them. They recorded however a tendency for worse motor UPDRS scores in fluoxetine group, but this was not statistically significant. One patient in the rTMS plus placebo group dropped out.

*c) Barone et al., 2006* (20)

Study description: Barone et al. (2006) conducted a randomized study in order to compare the antidepressant effect of sertraline to that of pramipexole in 67 patients with Parkinson’s disease. The participants were diagnosed with major depression according to the DSM-IV criteria. The authors did not mention whether demented patients were excluded. The trial duration was 12 weeks. The primary outcome measure was the change in the HDRS total score. The authors also assessed the response rate in each group typically defined as in other studies. Regarding methodological quality, the main limitation of the study was that the two treatments were given on an open-label basis. The authors note that the outcome assessment was performed by a trained rater blinded to treatment assignment.

Efficacy: In both groups the total HDRS scores decreased over time and there was a marginally non-significant trend in favour of pramipexole (p=0.055). The response rate in the sertraline group was 47% (16/34%) versus 70% (23/33) in the pramipexole group, a result which again was marginally non-sigificant (p=0.08).

Safety: Side effects were more common in the sertraline group (24% vs. 9% in the pramipexole group) and included vertigo, nausea, anxiety, abdominal pain, diarrhea, asthenia and tremor. Seven patients withdrew from sertraline treatment (21%) vs. one patient from pramipexole treatment (3%).
