# Supplementary material for: Efficacy and acceptability of selective serotonin reuptake inhibitors for the treatment of depression in Parkinson's disease: a systematic review and meta-analysis of randomized controlled trials
Source: BMC Neurol. 2010 Jun 21;10:49. doi: 10.1186/1471-2377-10-49 (PMC2903535; doi:10.1186/1471-2377-10-49)
Supplement: Additional file 3 — Details of the comparison between SSRIs and placebo using the continuous outcome. [file 1471-2377-10-49-S3.DOC]

**Additional File 3**

Paper: Skapinakis et al. Efficacy and acceptability of selective serotonin reuptake inhibitors for the treatment of depression in Parkinson’s Disease: Systematic review and meta-analysis of randomized controlled trials.

**Comparison 01: SSRI vs. placebo: Meta-Analysis of the continuous outcomes**

For this analysis we included 5 studies [14, 19, 21, 25, 26]. The study by Devos et al. (22) was excluded because the authors, due to the non-normal distribution of scores, have reported medians and quartiles only. We tried to impute means and standard deviations (SDs) using all the available data presented in the paper (although this is not correct due to violation of the normality assumption), this resulted in unrealistically very small values for SDs. Therefore, this study could not be used in the current analysis.

For the remaining five studies, the standardized mean difference using the Hedges’ g estimate was -0.13 (95% CI: -0.43 – 0.17, p=0.40) with no evidence of heterogeneity (Q= 2.71 with 4 degrees of freedom, p=0.61). A forest plot is shown below:
